# Anatomical Shaping for Zirconia Custom Implant Abutment to Enhance Anterior Esthetic: A Clinical Technique

**DOI:** 10.1155/2020/8857410

**Published:** 2020-11-05

**Authors:** Mohammed Alshehri, Mohammed Alghamdi, Abdullah S. Alayad

**Affiliations:** ^1^King Saud University Medical City, King Saud University, Riyadh, Saudi Arabia; ^2^Restorative Dental Sciences Department, College of Dentistry, King Saud University, Riyadh, Saudi Arabia

## Abstract

Abutments are used in dentistry to attach dental crowns to dental implant. Currently, zirconia custom abutment is the one which is mostly used in restorations, since it offers several advantages, especially better esthetics and prevention from infection. Several innovations are done in the implant designs and procedures to achieve better esthetics. Computer-aided design & computer-aided manufacturing (CAD/CAM) system is widely used innovative technology in dentistry. This technology offers custom implants that help to achieve better esthetics and good internal fit. This procedure used a novel technique of anatomical modification of the final abutment incisal edge from straight anatomical edge to irregular one with a mamelon-incisal effect to enhance esthetic, shade matching, and anatomical replication of incisal structure that resembles the natural incisor. Usually, dental technicians will perform facial and incisal cut-back and apply porcelain layers to the crown in order to reproduce the translucency and the other optical effects that most closely match that of natural dentin and enamel, especially at the incisal edge. These optical effects will make the prosthetic crown look more natural and esthetically pleasant. By this presented technique will help the dental technician to achieve highly esthetic crown with completely digital workflow without the need for porcelain layering. The procedure was also followed up to 3, 6, and 12 months after the surgery and found no complications or complaints from the patient and esthetically satisfied.

## 1. Introduction

Abutments are part of the implant prosthetic system which is used in dentistry to connect the implant crowns to dental implant [1]. They are broadly classified into two categories: stock abutment and custom or patient-specific abutment [2]. Stock implant abutments were economical and user friendly and can be used for implants at both tissue and bone level. Poor hygiene, poor esthetics, and poor alignment with angled implants led to the emergence of custom abutments [3]. In fact, new CAD/CAM systems registered a constantly increasing use in dentistry. This technology allows a completely digital workflow, from impression to final framework, and the materials used showed excellent mechanical properties [4] and good internal fit [5] which make it possible to create patient-specific abutments that are both strong and esthetic. Custom-made abutments can be fabricated by three techniques: lost wax technique, CAD/CAM, and 3D printing. Also, there are different materials that can be used to fabricate custom abutments, gold alloy, titanium, zirconia, and base metal alloy. Each one of these materials has advantages and disadvantages. Metal abutments, which are versatile and robust, can be used anywhere in the mouth, but esthetics is compromised when used in the anterior maxilla. Zirconia abutments, which are composed of ceramic, can be placed anywhere in the mouth, but are most advantageous, when used in the esthetic zone [6]. Besides its excellent strength and esthetic characteristics, zirconia exhibits hygienic properties comparable to titanium. Zirconia abutments can be layered by pink porcelain directly, when necessary, to approximate the color of the surrounding soft tissue. Zirconia has high strength and excellent biocompatibility, along with esthetics [3, 7–10]. The density and strength of zirconia are higher than those of titanium [11]. One main disadvantage of using zirconia abutment in direct internal connection with titanium abutment was internal damage to the titanium implant connection with abutment due to titanium wear that results in releases of titanium particle to adjacent soft tissue causing unesthetic greyish discoloration and screw loosening as a result of compromised abutment fit [12, 13]. For these reasons, *zirconia* “*hybrid*” *abutments* are prescribed more nowadays, where a zirconia body is luted in the laboratory to a short titanium connection feature—sometimes referred to as a titanium base to provide a titanium-to-titanium interface with the implant platform. These abutments are compatible with the same abutment screws used in titanium abutments. The titanium portion can also be color-coded during the manufacturing process, providing a visual cue to help immediately to identify the matching implant platform.

Esthetics is a very important aspect in implant dentistry to satisfy the patients, especially for implant-supported restorations in the anterior maxilla. However, achieving good esthetics in the anterior maxilla is difficult because of the complex anatomy. The ultimate aim of placing a dental implant is to anatomically restore the missing teeth, which can maximize the esthetic and restore function for a long duration [14]. Presurgery planning, suitable development of the site, 3D implant positioning, soft tissue management, provisionalization, and esthetic prosthetic management are the prerequisites for placing an esthetically pleasing implant [15]. All these steps have to be planned to meet patient's expectations.

Enhancement of the site following the extraction of tooth and preserving alveolar ridge is done to create appropriate hard and soft tissue volume so that implants will fit better [3]. Custom implants allow customizing cervical margins so that they are in a position to shape fitting the natural tooth root with better angulation. All these are done for better esthetic outcomes. A novel method for improving esthetic is to create mamelon and incisal configuration on implant abutment. The focus of this clinical report is to explore whether the mamelon and incisal configuration is successful in zirconia abutment with IPS™ e.Max CAD, for its regular use.

## 2. Clinical Report

A 28-year-old female patient presented herself to the dental clinic for evaluation of the restorability of the maxillary right central incisor. On clinical and radiographic evaluation, there was an endodontically treated tooth, associated with external cervical resorption. There was not much significant history about the patient and no contraindication for dental treatment. Periodontal examination using a double-ended, color-coded periodontal probe (Hue–Friedy, Hu-Friedy Mfg. Co., LLC, Chicago, USA) revealed thick gingiva, and on probing, it was free up to 3 mm with mild bleeding. Smile analysis showed high smile line and the gingival margin was visible in full smile. Diagnostic impressions were made with irreversible hydrocolloid (alginate impression, Henry Schein, New York, NY, USA), and bite registration was done using vinyl polysiloxane material (Imprint™ Bite, 3M ESPE, Seefeld, Germany).

The impressions and bite registration were sent to the dental laboratory for diagnostic cast fabrication and mounting. Combining data from all diagnostic findings, the tooth was rendered nonrestorable. She was very concerned about her esthetics and was willing for the earliest possible replacement of teeth. Hence, decision was made for extraction, debridement of the site, immediate implant placement, and immediate loading.

## 3. Findings

Presurgical radiographic evaluation was done with a panoramic radiograph (KaVo OP 3D™ Pro for 2D, Brea, CA, USA), and the length and width of the available bone were measured using Denta Scan. Accordingly, dental implant size and length were chosen. The patient was administered 2 grams of amoxicillin one hour before surgery. After injecting lidocaine HCl 2% with adrenaline 1 : 100000 for local anesthesia, the maxillary right central incisor was removed atraumatically. Thorough debridement of the extraction socket was done under saline wash. Periodontal probing revealed an intact cortical plate. Drilling was done sequentially to prepare the osteotomy site. Dental implant (4.3 × 13 mm) platform 3.4 mmD (Implant Direct™ InterActive System, Thousand Oaks, California, USA) was inserted in the drilled osteotomy site with an insertion torque of 45 NCm, and adequate primary stability was obtained [16]. Postoperatively intraoral periapical radiograph was taken to confirm the position of the implant. A titanium temporary abutment was attached to the implant, and a provisional crown shell was connected to the abutment by using flowable composite (Filtek™ Z350 XT Flowable, 3M ESPE, Seefeld, Germany). The temporary abutment was removed from the patient's mouth, and the emergence profile was corrected by adding more flowable composite followed by finishing and polishing. After that, the temporary crown was attached to the implant and relieved from occlusion. An antibiotic 500 mg amoxicillin tablet tid for 5 days and analgesic ibuprofen 400 mg·prn were prescribed postoperatively. After 3 months, the provisional crown was removed and the final impression was done by using a chairside custom-made impression post that mimics the cervical part of the provisional restoration to support soft tissue contours after provisional removal. Impression was sent to the laboratory for the fabrication of a single-piece zirconia hybrid custom abutment.

**Figure 1:**
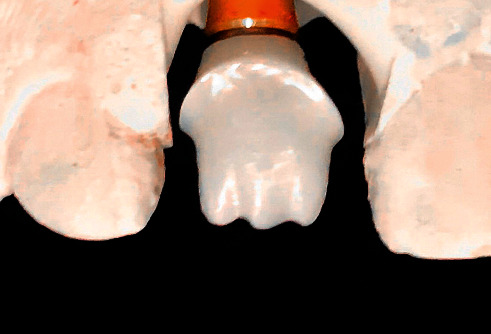
Zirconia custom abutment with mamelon-incisal effect.

**Figure 2:**
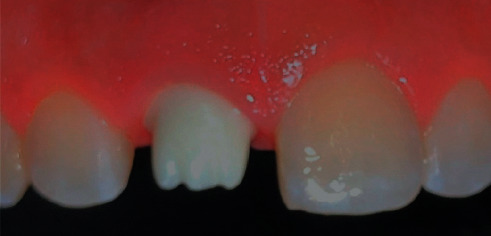
Zirconia custom abutment (final) try-in in the patient's mouth.

**Figure 3:**
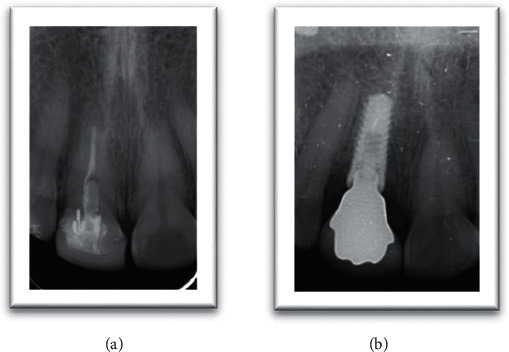
Preoperative and postoperative radiographs.

**Figure 4:**
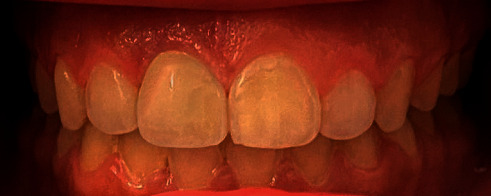
Final photograph after cementation.

The custom abutment was attached to the implant body and prepared for parallelism, adequate space, and finish line position in relation to the gingival margin. The final abutment incisal edge was modified from straight nonanatomical edge to irregular one with a mamelon-incisal effect to enhance esthetic appearance, shade matching, and for anatomical replication of incisal structure (Figures 1 and 2). The custom abutment was sent to the laboratory for the fabrication of lithium disilicate glass-ceramic crown (IPS™ e.Max CAD, Ivoclar Vivadent AG, Schaan, Liechtenstein) and abutments cementation to the crown (Figure 3). The final cemented screw-retained crown was tried in the patient's mouth and then torqued to 30 NCm. A sterilized piece of Teflon tape was placed in the screw access hole above the screw head and then the access hole was closed by composite resin (Filtek™ Z350 XT Universal, 3M ESPE, Seefeld, Germany), occlusion was rechecked again, and final finishing and polishing were done to composite restoration. Follow-up was done after 3, 6, and 12 months' intervals. The implant restoration was successful with no complaints from the patient and was esthetically pleasing (Figure 4).

## 4. Discussion

The current primary need of the patients coming for dental restoration is to achieve better esthetics and faster healing rate. This needs thorough presurgical planning, accurate three-dimensional implant placement, careful soft tissue management, and proper provisional restorations [3]. Innovative procedures and designs are needed to improve the implant-supported restorations for better esthetics, biological compatibility, and durability to satisfy the patients. In this clinical report, a novel technique of modifying the incisal edge of the abutment was introduced from straight nonanatomical edge to irregular one with a mamelon-incisal effect to enhance esthetic and shade matching. This technique seems to be successful after following up for 12 months.

Implant restoration in the anterior maxillary area needs complex procedures to achieve esthetics because of the complex anatomy. The natural tooth has complex structures of enamel and dentine that are able to collect and distribute light within the tooth, with both enamel prisms and dentinal tubules acting as optical fibers. As light passes through the enamel, it is modified by the thickness and translucency of the enamel, which allows dentine, the primary source of color, to shine through [17]. For a dental technician to be able to reproduce these optical effects that mimic the natural teeth, he will need to perform a cut-back in the prosthetic crown and apply multiple layers of porcelain. This technique will need a high level of skills and knowledge in addition to the extra time for porcelain firing. Creation of a custom zirconia abutment with irregular mamelon will give us the optical effect of mamelon under a translucent crown. This technique will allow the technician to create an esthetically pleasant crown with a completely digital workflow which will facilitate the crown fabrication procedure and saving time.

Using a single-piece zirconia hybrid custom abutment with titanium base to connect the crown to the implant at an esthetic zone will solve esthetic problems and eliminate the complication that may result from using completely zirconia abutment such as unesthetic greyish discoloration and screw loosening due to internal damage to the titanium implant connection with abutment [12, 13].

Extraoral cementation of the crown will ensure complete elimination of excess cement, therefore reducing the possibility of infection as a result of retained excess cement during intraoral cementation [18, 19].

Immediate implant placement and immediate loading which have already been tried previously have high success rate, faster healing time, good osseointegration, and better esthetics [20–23].

However, there are many advantages of this dental implant, but it also has some limitations. Minimizing undesirable stress concentrations, computer-milled custom abutments must fit accurately [24, 25]. The effect of mamelon presence on stress concentration and distribution has to be confirmed scientifically with mechanical tests and experiments. Loss of retention due to short titanium base and weak bonding strength between cement and zirconia is also another complication that may happen in future so a longer follow-up is needed [26]. Also in some cases with high esthetic demands, the technician will need to do some staining or to use the porcelain layering technique to match the esthetic characteristics especially in matching one single maxillary central incisor to a highly characterized natural maxillary central incisor. Another factor that resists people to go for this procedure is high cost. Custom implants are more costly that they are not affordable by a common person. Further improvement needs to be done to make this procedure accessible to everyone [27].

## 5. Conclusion

Several innovations have been identified in the design and procedure of implant restoration to achieve better esthetics. This report has modified the final abutment incisal edge from straight nonanatomical edge to irregular one with a mamelon-incisal effect to enhance esthetic appearance, shade matching, and anatomical replication of incisal structure.

## Data Availability

The data will be available for review from the corresponding author on request.
